# The Calculation of Language Lateralization Indices in Post-stroke Aphasia: A Comparison of a Standard and a Lesion-Adjusted Formula

**DOI:** 10.3389/fnhum.2016.00493

**Published:** 2016-10-13

**Authors:** Aimee Dietz, Jennifer Vannest, Thomas Maloney, Mekibib Altaye, Jerzy P. Szaflarski, Scott K. Holland

**Affiliations:** ^1^Department of Communication Sciences and Disorders, University of CincinnatiCincinnati, OH, USA; ^2^Pediatric Neuroimaging Research Consortium, Cincinnati Children’s Hospital Medical CenterCincinnati, OH, USA; ^3^Division of Biostatistics and Epidemiology, Cincinnati Children’s Hospital Medical CenterCincinnati, OH, USA; ^4^Department of Neurology, University of Alabama at BirminghamBirmingham, AL, USA; ^5^Department of Radiology, Cincinnati Children’s Hospital Medical CenterCincinnati, OH, USA

**Keywords:** aphasia, fMRI, LI, language lateralization index, ROI, region of interest

## Abstract

**Background:** The language lateralization index (LI) is a valuable tool in functional magnetic resonance imaging (fMRI) research, especially in people with post-stroke aphasia. However, there is inconsistent consideration for the overlap of lesions with regions of interest (ROIs). The purpose of this study was to determine whether standard LI (SLI) and lesion-adjusted LI (LALI) formulae generate different LI values and language lateralization classification for people with post-stroke chronic aphasia.

**Methods:** SLI and LALI were calculated for an event-related (overt) verb generation task in an anterior and a posterior language ROI. Twelve people with aphasia due to a single left-hemispheric infarct (11 right-handed; 1 left-handed; 77.2 ± 41.7 months post-stroke) were included (eight females; 57 ± 8.88 years). Spearman correlation coefficients and intraclass correlation coefficients were calculated to determine the relationship of the LI values generated by the SLI and the LALI formulas. Fischer’s exact test and a weighted Cohen’s Kappa determined the difference in language lateralization classification and agreement in the classification. Spearman correlation was used to examine the relationship between the difference in lateralization values produced by the LALI and SLI calculations with (1) lesion size, (2) the percentage of lesion overlap in each ROI, and (3) aphasia severity.

**Results:** The two calculation methods were highly correlated and produced similar LI Values, yet yielded significantly different classification for language lateralization. Further, a more leftward LI resulted from application of the LALI formula in 10 participants, in either the anterior ROI (*n* = 3) or the posterior ROI (*n* = 7). Finally, for the posterior ROI only, significant correlations were revealed between the two calculation methods and the (1) lesion size and (2) percent of overlap with the ROI.

**Discussion:** While both approaches produce highly correlated LI values, differences in activation lateralization between formulas were observed, including changes in lateralization classification. Examination of the issues raised in the current investigation need to be replicated with a larger sample to determine the utility of a LALI formula in predicting behavioral performance; the findings may have implications for understanding and interpreting fMRI data of people with post-stroke aphasia.

## Introduction

Since aphasiologists are interested in determining the influence of intervention on the neural reorganization of language function, the language lateralization index (LI) is a valuable outcome measure in functional magnetic resonance imaging (fMRI) research because it provides a way to quantify the contribution of left hemispheric perilesional tissue assuming responsibility for language functions relative to the right hemispheric homolog(s). Although an exhaustive review of the decades-long “left versus right hemisphere debate” in post-stroke aphasia recovery is beyond the scope of this paper, a brief summary is warranted [interested readers are referred to Berthier et al. (2011) for a more extensive review]. A plethora of data suggests that at least initially, the right hemisphere assumes responsibility for performing language tasks formerly managed by the left hemisphere. Then, those who recover best, demonstrate a shift of language functions back to the left hemispheric, perilesional regions (Saur et al., 2006, 2010; Szaflarski et al., 2013). However, the right hemisphere may play a more critical role in recovery than previously acknowledged (Crosson et al., 2009; Fridriksson et al., 2012; Faseyitan et al., 2013); this may be especially true for people with aphasia who have larger lesions and less residual healthy tissue capable for absorbing the responsibility for linguistic functions (Heiss and Thiel, 2006; Crosson et al., 2009; Berthier et al., 2011). More recently, though, rather than debating the importance or prominence of the left versus the right hemisphere, many scientists have adopted the notion that both hemispheres play a critical role in post-stroke language recovery. Thus, the central problem now faced by aphasiologists is to determine under which circumstances, and for which language functions, left and right hemisphere networks are best suited to take command (Crosson et al., 2007; Turkeltaub et al., 2011; Faseyitan et al., 2013). Further, neuroimaging scientists are beginning to identify treatment responders based on neural reorganizational patterns observed during carefully designed language-based fMRI paradigms that reflect the skill trained during treatment (Fridriksson et al., 2006; Thompson et al., 2010). For these reasons, it is imperative that the language LI values are reliable, and that researchers and clinicians alike understand the methodological factors that can influence derivation of the LI value.

A multitude of factors are known to influence the directionality of the LI value. According to Seghier (2008), these can be categorized as follows: (1) quantification of the relative contribution of the left and right hemisphere, (2) regions of interest (ROI) selection, (3) variability and reproducibility of LI values, (4) reliance on statistical thresholding, (5) thresholding for hemispheric dominance, (6) task selection, (7) contrast (or control) conditions, and a catch-all classification, and (8) “other considerations” (pp. 599). The current study seeks to provide evidence to clarify the importance of how the contribution of the left and right hemispheres is quantified specifically, in the post-stroke population. Presently, there is inconsistent attention in the literature regarding the influence of lesion size and lesion overlap with ROIs on the LI values reported in language recovery studies. A frequently used standard LI (SLI) formula is calculated by counting the number of voxels that activate above the median *z* score of the ROI and dividing by the total number voxels located in the ROI (lesioned or not), such that the left and right ROIs are identical in size (Davis et al., 2006; Crosson et al., 2009; Szaflarski et al., 2013). This method seems to naturally bias the LI values rightward since lesioned voxels, which, presumably, cannot be activated, are included in the left-hemispheric ROIs. An alternative to this approach is to apply a lesion-adjusted LI (LALI) formula; one, which accounts for the lesion in some manner. While some researchers have employed LI formulas that address this concern (Bonakdarpour et al., 2007; Sebastian and Kiran, 2011) the difference in the derived lateralization values between a standard and a lesion-adjusted method has not been examined. As a preliminary step toward understanding the implications of this tendency in the literature, the purpose of this study was to evaluate whether a SLI and an LALI method (1) generate different LI values and (2) different categorization of language lateralization classification in two language ROIs for 12 patients with stroke-induced aphasia on a verb generation task.

To address the questions posed in this study, we used an event-related verb generation task and a sparse acquisition approach, which allows participants to hear the auditory prompts without scanner noise, and provide overt verbal responses while avoiding head motion during image acquisition (Schmithorst and Holland, 2004; Allendorfer et al., 2012a). This approach has been shown to better detect task-related activation patterns than a standard boxcar fMRI (Schmithorst and Holland, 2004; Huettel, 2012). We examined the difference in SLI and LALI values in two anatomical ROIs, which encompass broad regions of the language network and have been shown to capture perilesional activation that may occur as a result of post-stroke recovery and language-related reorganization (Allendorfer et al., 2012a,b) (see “Materials and Method” for more details).

Specifically, we asked the following questions:

1. Do the LIs derived by the SLI and LALI formulas:
  (a) Generate different LI values?
  (b) Categorize language lateralization in people with post-stroke aphasia differently.
2. What is the relationship between LALI and SLI values and:
  (a) Left hemisphere lesion size?
  (a) Percent overlap between ROI and left hemisphere lesion?
3. What is the relationship between the LALI and SLI values and aphasia severity?

We hypothesized that would be a significant difference in the classification of language lateralization when considering the two methods. Further, we hypothesized a positive correlation between lesion size and the degree of overlap between the lesion and the ROI on the difference in LI values. That is, the larger the lesion and the greater the overlap with the ROI, the more likely the SLI and LALI results would differ. Lastly, since our primary goal was to examine the whether the SLI and LALI calculation methods differed in resultant LI values/language lateralization classification, the final research question, regarding aphasia severity, was exploratory in nature.

## Materials and Methods

This study was carried out in accordance with the recommendations of the University of Cincinnati and Cincinnati Children’s Hospital and Medical Center Institutional Review Board, with written informed consent from all subjects.

### Participants

The participants included 12 people with chronic, post-stroke aphasia (single left middle cerebral ischemic infarct) who participated in a larger treatment study. Prior to the stroke, all but one participant were right-handed, according to the Edinburgh Handedness inventory (Oldfield, 1971). Further, all participants were native speakers of American English, had at least a high school education, and reported a no history of psychiatric substance abuse. Prior to enrolling in the study, all participants reported normal or corrected vision, passed a visual field cut screening, and a passed a hearing screening in at least one ear at (i.e., 40 db HL 1000, 2000, and 4000 Hz). **Table 1** provides a summary of the participants’ demographic data.

**Table 1:** Demographic and linguistic profile of participants.

ID	Gender	Ethnicity	Age	Number lesioned voxels	MPO^a^	Level of education	Aphasia type^b^	Aphasia quotient^b^
1	Female	Caucasian	57	18727	79	Some college	Global	40.8
2	Male	Caucasian	61	30570	91	Bachelor’s	Broca’s^c^	68.6
3	Female	African amer.	58	1583	48	Bachelor’s	Anomic	89.2
4	Female	Caucasian	47	14616	124	Bachelor’s	Broca’s^c^	48.9
5^d^	Female	Caucasian	60	12950	105	Some college	Conduction	74.0
6	Male	Caucasian	59	14009	73	Master’s	Anomic	68.9
7	Male	Caucasian	47	10989	69	High school	Wernicke’s	55.5
8	Male	Caucasian	71	14066	69	Master’s	Broca’s^c^	62.7
9	Female	Caucasian	63	17705	170	Bachelor’s	Conduction	71.2
10	Female	Caucasian	57	7849	16	High school	Wernicke’s	37.6
11	Female	Caucasian	39	18807	44	Some college	Anomic	82.4
12	Male	Caucasian	66	30110	38	Bachelor’s	Broca’s^c^	36.7

*^a^Months post-onset; ^b^Western Aphasia Battery-Revised (Kertesz, 2006) used to determine type and severity, Aphasia quotient out of 100; ^c^Apraxia of Speech present based on clinical judgment; ^d^Only left-handed participant; all others right-handed; handedness based on the Edinburgh Handedness Inventory (i.e., score ≥ 50 = right-handed) (Oldfield, 1971)*.

### FMRI Equipment and Methods

A 3.0 Philips Achieva Whole Body MRI/MRS system allowed acquisition of structural and fMRI scans. The following parameters were used to capture high-resolution T1-weighted anatomical images: TR/TE = 8.1/3.7 ms, FOV 25.6 × 25.6 × 19.2 cm, matrix 256 × 256, slice thickness = 1 mm. A sparse acquisition [see “Event-Related Verb Generation Task (ER-VGT)” for details] approach was used for each trial: MRI silence occurred for the first 6 s to allow stimuli presentation and participant response; followed by 6 s (three image volumes) of fMRI data acquisition during the height of the hemodynamic response (Schmithorst and Holland, 2004). The fMRI scanning was performed with the following parameters: TR/TE = 2000/38 ms, FOV 24.0 × 24.0 cm, matrix 64 × 64, slice thickness = 4 mm, SENSE factor = 2. This resulted in a voxel size of 3.75 × 3.75 × 4 mm and 32 axial slices. FMRI task was developed in DirectRT (version 2012^1^) and presented using an Avotec audio-visual system.

#### Event-Related Verb Generation Task (ER-VGT)

In this study, we employed a well-documented event related verb generation task that is recognized for its sensitivity to identify language-related areas in healthy controls and patients with post-stroke aphasia (Allendorfer et al., 2012a,b). Participants viewed a Ready screen for 4 s. Then, they completed 15 alternating trials each of: (1) covert verb generation, (2) overt verb generation, and (3) overt noun repetition, with each of the 45 trials lasting 12 s. The first 6 s of each trial began with MRI silence, followed by 6 s of fMRI acquisition. In lieu of written instructions, due to the known comprehension challenges experienced by people with aphasia, the participants were presented with a pictorial instructions during the first 1 s of MRI silence. Following pictorial instruction, the remaining 5 s of MRI silence included an auditory presentation of a concrete noun (e.g., “cookie”) via headphones. The pictorial instruction cued the participant to produce associated verbs (e.g., bake, eat, ice, etc.) either (1) to themselves (covert generation) or (2) aloud (overt generation), or to (3) repeat the noun aloud (i.e., “cookie, cookie, cookie”; over repetition). The final 6 s of each trial concluded with fMRI data acquisition.

Prior to entering the scanner, all participants practiced the ER-VGT and were able to provide at least a least one correct response to each of the aforementioned three ER-VGT conditions. That is they were able to speak an appropriate response for the overt verb generation and overt noun repetition trials; and made no overt responses during the covert verb generation trials. This is in line with other reports of the ER-VGT task performance by people who have aphasia (Allendorfer et al., 2012a). People with aphasia can learn the task; however, they typically produce fewer verb productions during the overt verb generation task when compared to healthy controls. During the scan, participants were monitored by in-scanner microphone on each trial to ensure that the participants responded as instructed. In instances where the participant confused the instructions, the sequence was stopped and the patient was reinstructed (Participants 3, 6, 7, 8, 9, 10, 11, and 12). With the exception of Participant 12, all participants were able to respond, or attempt to respond, appropriately during each condition after reinstruction.

The ER-VGT task involves several speech and linguistic functions; with auditory processing being required for all tasks. The contrast of overt > covert generation isolates articulatory ability, or motor aspects of speaking, while controlling for recognition of the auditory noun, and noun-verb semantic associations needed to generate the verb responses. The contrast of overt verb generation > overt repetition isolates the noun-verb semantic association process while controlling for recognition of the auditory noun and articulatory/motor aspects of generating an overt response. In this study, we used a general linear model approach to identify voxels that were more active in the overt verb generation > overt repetition contrast.

### Research Design and Data Analyses

**FIGURE 1:**
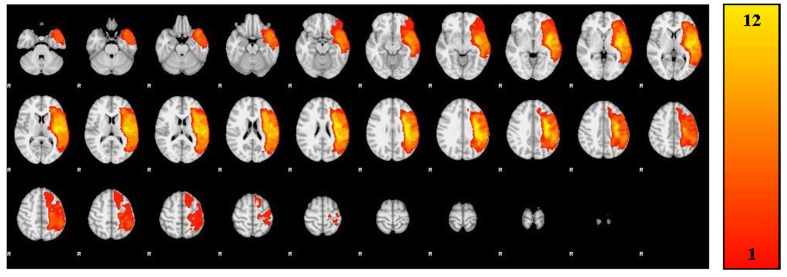
**Lesion overlay of all 12 participants**.

A trained neuroanatomist (JPS) manually traced each participant’s lesion on their T1-weighted anatomical image via Analysis of Functional Neuroimages (AFNI) (Cox, 2012). **Figure 1** and **Table 2** depict the lesion overlay and lesion location, respectively, for all 12 participants. Next, spatial normalization to MNI standard space and motion correction were completed using the Oxford FMRIB software library (FSL) (Smith et al., 2004; Woolrich et al., 2009; Jenkinson et al., 2012). For motion correction, the MCFLIRT tool was used; outlying frames were detected using RapidART, a part of Nipype (Gorgolewski et al., 2011), with a composite motion threshold of 2 mm and Z-intensity threshold of 3. The six motion parameters (three translation, three rotation) were used as regressors in the design model along with any outlying frames; mean displacement was less than 1.5 mm for each participant (*Mean* = 0.33; *SD* = 1.44).

**Table 2:** Description of lesions for the 12 participants.

ID	Number lesioned voxels	Left hemisphere lesion location
1	18727	Inferior frontal gyrus; pars triangularis; subcallosal cortex; angular gyrus; frontal medial cortex; middle temporal gyrus (temporooccipital region); central opercular cortex; frontal operculum cortex; superior (posterior), middle (anterior), and inferior gyrus (anterior and posterior); postcentral gyrus; Heschl’s gyrus (includes H1 and H2); lateral occipital cortex; inferior division amygdala; inferior temporal gyrus; temporooccipital part; frontal orbital cortex; insular cortex; temporal fusiform cortex (anterior; posterior); temporal pole; middle frontal gyrus; supramarginal gyrus ( posterior division); parietal operculum cortex; lateral occipital cortex (superior division); planum temporale; inferior frontal gyrus; pars opercularis; middle temporal gyrus (posterior division); planum polare; frontal pole; superior temporal gyrus (anterior division); supramarginal gyrus (anterior division); precentral gyrus
2	30570	Inferior frontal gyrus, pars triangularis; cuneal cortex; subcallosal cortex; angular gyrus; frontal medial cortex; superior parietal lobule; middle temporal gyrus, temporooccipital part; central opercular cortex; superior frontal gyrus; cingulate gyrus, anterior division; supracalcarine cortex; lingual gyrus; frontal operculum cortex; superior temporal gyrus (posterior division); inferior temporal gyrus (anterior division); middle temporal gyrus (anterior division); inferior temporal gyrus (posterior division); postcentral gyrus; Heschl’s gyrus (includes H1 and H2); juxtapositional lobule cortex (formerly supplementary motor cortex); lateral occipital cortex ( inferior division); cingulate gyrus (posterior division); parahippocampal gyrus (anterior division); inferior temporal gyrus, temporooccipital part; frontal orbital cortex; insular cortex; temporal fusiform cortex, anterior division; temporal pole; middle frontal gyrus; temporal fusiform cortex (posterior division); supramarginal gyrus ( posterior division); parietal operculum cortex; lateral occipital cortex (superior division); occipital fusiform gyrus; precuneus cortex; planum temporale; inferior frontal gyrus, pars opercularis; middle temporal gyrus (posterior division); intracalcarine cortex; planum polare; frontal pole; superior temporal gyrus (anterior division); supramarginal gyrus (anterior division); precentral gyrus
3	1583	Frontal operculum cortex; inferior frontal gyrus, pars triangularis; parietal operculum cortex; central opercular cortex; putamen; frontal orbital cortex; inferior frontal gyrus, pars opercularis; middle frontal gyrus; frontal pole; supramarginal gyrus (anterior division); precentral gyrus; postcentral gyrus
4	14616	Inferior frontal gyrus, pars triangularis; angular gyrus; superior parietal lobule; middle temporal gyrus, temporooccipital part; central opercular cortex; frontal operculum cortex; superior temporal gyrus (posterior division); middle temporal gyrus (anterior division); postcentral gyrus; Heschl’s gyrus (includes h1 and h2); frontal pole; frontal orbital cortex; insular cortex; temporal fusiform cortex, anterior division; temporal pole; middle frontal gyrus; temporal fusiform cortex (posterior division); supramarginal gyrus (posterior division); parietal operculum cortex; lateral occipital cortex (superior division); planum temporale; paracingulate gyrus; inferior frontal gyrus, pars opercularis; middle temporal gyrus (posterior division); planum polare; superior temporal gyrus (anterior division); supramarginal gyrus (anterior division); precentral gyrus
5	12950	Superior parietal lobule; angular gyrus; middle temporal gyrus, temporooccipital part; temporal occipital fusiform cortex; central opercular cortex; amygdala; lingual gyrus; frontal operculum cortex; superior temporal gyrus (posterior division); inferior temporal gyrus (anterior division); middle temporal gyrus (anterior division); inferior temporal gyrus (posterior division); postcentral gyrus; Heschl’s gyrus (includes H1 and H2); lateral occipital cortex (inferior division); cingulate gyrus (posterior division); inferior temporal gyrus, temporooccipital part; frontal orbital cortex; insular cortex; temporal fusiform cortex (anterior and posterior division); temporal pole; middle frontal gyrus; supramarginal gyrus (posterior division); parietal operculum cortex; lateral occipital cortex (superior division); occipital fusiform gyrus; precuneus cortex; planum temporale; parahippocampal gyrus (posterior division); middle temporal gyrus (posterior division); cuneal cortex; planum polare; frontal pole; superior temporal gyrus (anterior division); supramarginal gyrus (anterior division); precentral gyrus
6	14009	Inferior frontal gyrus, pars triangularis; planum polare; central opercular cortex; superior frontal gyrus; frontal operculum cortex; superior temporal gyrus (posterior division); inferior temporal gyrus, anterior division; middle temporal gyrus (anterior division); inferior temporal gyrus, posterior division; postcentral gyrus; Heschl’s gyrus (includes H1 and H2); juxtapositional lobule cortex (formerly supplementary motor cortex); parahippocampal gyrus (anterior division); frontal pole; frontal orbital cortex; insular cortex; temporal fusiform cortex (anterior division); temporal pole; middle frontal gyrus; temporal fusiform cortex (posterior division); supramarginal gyrus (posterior division); parietal operculum cortex; planum temporale; paracingulate gyrus; inferior frontal gyrus, pars opercularis; middle temporal gyrus (posterior division); superior temporal gyrus (anterior division); supramarginal gyrus (anterior division); precentral gyrus
7	10989	Angular gyrus; superior parietal lobule; middle temporal gyrus, temporooccipital part; central opercular cortex; supracalcarine cortex; superior temporal gyrus (posterior division); middle temporal gyrus (anterior division); inferior temporal gyrus (posterior division); cuneal cortex; Heschl’s gyrus (includes H1 and H2); postcentral gyrus; juxtapositional lobule cortex (formerly supplementary motor cortex); parahippocampal gyrus (anterior division); inferior temporal gyrus, temporooccipital part; hippocampus; insular cortex; temporal pole; temporal occipital fusiform cortex; supramarginal gyrus (posterior division); parietal operculum cortex; lateral occipital cortex (superior division); precuneus cortex; planum temporale; parahippocampal gyrus (posterior division); middle temporal gyrus (posterior division); intracalcarine cortex; planum polare; temporal fusiform cortex (posterior division); supramarginal gyrus (anterior division); precentral gyrus
8	14066	Occipital fusiform gyrus; middle temporal gyrus (anterior division); inferior temporal gyrus (posterior division); intracalcarine cortex; Heschl’s gyrus (includes H1 and H2); lateral occipital cortex (inferior division); inferior temporal gyrus, temporooccipital part; frontal orbital cortex; insular cortex; temporal pole; middle frontal gyrus; temporal fusiform cortex (posterior division); supramarginal gyrus (posterior division); parietal operculum cortex; lateral occipital cortex (superior division); planum temporale; inferior frontal gyrus, pars opercularis; middle temporal gyrus (posterior division); postcentral gyrus; planum polare; frontal pole; superior temporal gyrus (anterior division); supramarginal gyrus (anterior division); precentral gyrus
9	17705	Inferior frontal gyrus, pars triangularis; angular gyrus; superior parietal lobule; middle temporal gyrus, temporooccipital part; central opercular cortex; superior frontal gyrus; cingulate gyrus (anterior division); frontal operculum cortex; superior temporal gyrus (posterior division); inferior temporal gyrus (anterior division); middle temporal gyrus (anterior division); inferior temporal gyrus (posterior division); postcentral gyrus; Heschl’s gyrus (includes H1 and H2); juxtapositional lobule cortex (formerly supplementary motor cortex); lateral occipital cortex (inferior division); cingulate gyrus (posterior division); parahippocampal gyrus (anterior division); inferior temporal gyrus, temporooccipital part; parahippocampal gyrus, posterior division; insular cortex; temporal fusiform cortex (anterior division); temporal pole; middle frontal gyrus; temporal fusiform cortex (posterior division); supramarginal gyrus (posterior division); parietal operculum cortex; lateral occipital cortex (superior division); precuneus cortex; planum temporale; paracingulate gyrus; inferior frontal gyrus, pars opercularis; middle temporal gyrus (posterior division); planum polare; superior temporal gyrus (anterior division); supramarginal gyrus (anterior division); precentral gyrus
10	7849	Inferior frontal gyrus, pars triangularis; angular gyrus; superior parietal lobule; middle temporal gyrus, temporooccipital part; central opercular cortex; frontal operculum cortex; superior temporal gyrus (posterior division); middle temporal gyrus (anterior division); inferior temporal gyrus (posterior division); postcentral gyrus; Heschl’s gyrus (includes H1 and H2); lateral occipital cortex (inferior division); inferior temporal gyrus, temporooccipital part; frontal orbital cortex; insular cortex; temporal pole; superior temporal gyrus (anterior division); supramarginal gyrus (posterior division); parietal operculum cortex; lateral occipital cortex (superior division); planum temporale; inferior frontal gyrus, pars opercularis; middle temporal gyrus (posterior division); planum polare; temporal fusiform cortex (posterior division); supramarginal gyrus (anterior division); precentral gyrus
11	18807	Inferior frontal gyrus, pars triangularis; planum polare; superior parietal lobule; central opercular cortex; superior frontal gyrus; cingulate gyrus (anterior division); frontal operculum cortex; superior temporal gyrus (posterior division); middle temporal gyrus (anterior division); postcentral gyrus; Heschl’s gyrus (includes H1 and H2); juxtapositional lobule cortex (formerly supplementary motor cortex); parahippocampal gyrus (anterior division); frontal pole; insular cortex; temporal pole; middle frontal gyrus; supramarginal gyrus (posterior division); parietal operculum cortex; precuneus cortex; planum temporale; paracingulate gyrus; inferior frontal gyrus, pars opercularis; superior temporal gyrus (anterior division); supramarginal gyrus (anterior division); precentral gyrus
12	30110	Cingulate gyrus (anterior division); supracalcarine cortex; frontal operculum cortex; superior temporal gyrus (posterior division); inferior temporal gyrus (anterior division); middle temporal gyrus (anterior division); inferior temporal gyrus (posterior division); postcentral gyrus; Heschl’s gyrus (includes H1 and H2); juxtapositional lobule cortex (formerly supplementary motor cortex); lateral occipital cortex (inferior division); cingulate gyrus (posterior division); parahippocampal gyrus (anterior division); inferior temporal gyrus, temporooccipital part; frontal orbital cortex; insular cortex; temporal fusiform cortex (anterior division); temporal pole; middle frontal gyrus; temporal occipital fusiform cortex; supramarginal gyrus, posterior division; parietal operculum cortex; lateral occipital cortex (superior division); occipital fusiform gyrus; precuneus cortex; planum temporale; parahippocampal gyrus (posterior division); inferior frontal gyrus, pars opercularis; middle temporal gyrus (posterior division); intracalcarine cortex; planum polare; frontal pole; superior temporal gyrus (anterior division); supramarginal gyrus (anterior division); precentral gyrus

*Lesioned areas defined via the Harvard Oxford atlas (Desikan et al., 2006)*.

#### Regions of Interest

**FIGURE 2:**
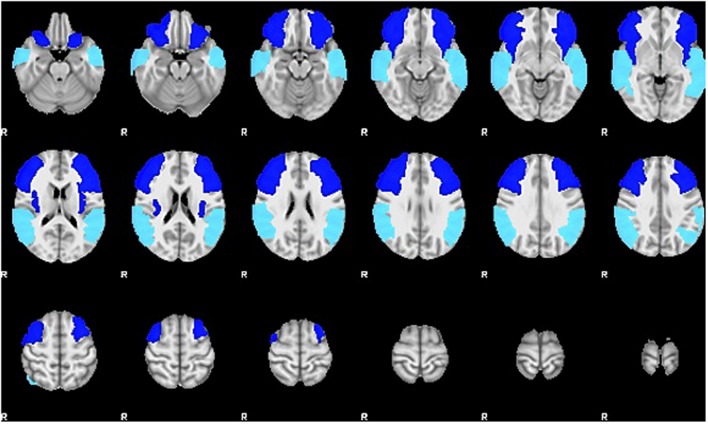
**Two anatomical ROIs created via the Automatic Anatomical Labelling atlas (AAL) (Tzourio-Mazoyer et al., 2002) in AFNI (Cox, 2012) used to calculate the standard and lesion-adjusted language lateralization indices**. The anterior ROI (royal blue) includes the inferior frontal gyrus, and contiguous regions of the middle frontal gyrus, precentral gyrus. The posterior ROI (turquoise) includes posterior superior and middle temporal gyri extending into inferior temporal gyrus (Allendorfer et al., 2012a,b).

For the current study, we examined the difference in SLI and LALI values in two anatomical ROIs created via the Automatic Anatomical Labelling atlas (AAL) (Tzourio-Mazoyer et al., 2002) in AFNI (Cox, 2012). The ROIs were based on previously described anterior and posterior language zones (Tillema et al., 2008). The anterior ROI, which corresponds to Broca’s area, includes inferior frontal gyrus, and contiguous regions of the middle frontal gyrus, precentral gyrus. The posterior ROI, which corresponds to Wernicke’s area, includes posterior superior and middle temporal gyri extending into inferior temporal gyrus (Allendorfer et al., 2012a,b). These regions, are depicted in **Figure 2** and were selected to include regions beyond Broca’s and Wernicke’s areas, proper. As such, these ROIs encompass broad regions of the language network, have been shown to capture perilesional activation that may occur as a result of post-stroke recovery and language-related reorganization (Allendorfer et al., 2012a,b). The sensitivity of these ROIs to detect language-related activation for noun-verb semantic associations (overt verb generation > overt noun repetition) during the ER-VGT was verified in a group of 16 right-handed people with post-stroke aphasia and a healthy control cohort matched for age-, gender, and pre-stroke handedness (Allendorfer et al., 2012a,b).

#### Thresholding and Language Lateralization Calculation Methods

**FIGURE 3:**
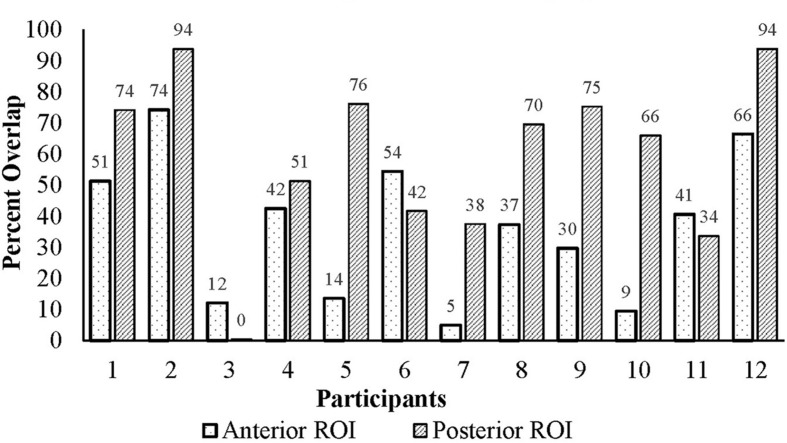
**Percent lesion/ROI overlap for all participants**.

The ROIs were applied to each participant’s functional data and mirrored to the right in MNI space. **Figure 3** illustrates the percent lesion/ROI overlap for each participant in current study. Thresholding for active voxels, for purposes of LI calculation, was performed on a single-participant basis. Specifically, voxels above the median *z*-score (overt verb generation > overt repetition) in each ROI for each participant were counted as active (Wilke and Schmithorst, 2006; Wilke and Lidzba, 2007). Next, the language LI was calculated using two methods: a SLI and a lesion-adapted LI formula as described below. The language lateralization classification schema for this study is based on the specific criteria used in our previous work with the verb generation task (Szaflarski et al., 2006, 2008, 2014; Allendorfer et al., 2012a,b), and that of others (Wilke and Lidzba, 2007). Specifically, right-lateralization was defined as LI < -0.1, and left-lateralization as >0.1; whereas values between -0.1 < LI ≤ 0.1 indicated bilateral, or symmetric language distribution.

##### Standard lateralization index (SLI)

The SLI formula (see below) applied in this study was calculated by counting the number of active voxels (those above the median *z*-score of the ROI). Then, we calculated the difference between the right and left ROIs divided by the total number of active voxels in the right and left ROIs (lesioned or not). The left and right ROIs are identical in size (Szaflarski et al., 2006, 2014; Wilke and Lidzba, 2007), and this calculation includes all voxels in left and right ROIs, including lesioned tissue that overlaps with the ROIs.

$$\frac{\left(\#\,L\,e\,f\,t\,\;A\,c\,t\,i\,v\,e\,\;V\,o\,x\,e\,l\,s-\#\,R\,i\,g\,h\,t\,\;A\,c\,t\,i\,v\,e\,\;V\,o\,x\,e\,l\,s\right)}{\left(\#\,L\,e\,f\,t\,\;A\,c\,t\,i\,v\,e\,\;V\,o\,x\,e\,l\,s+\#\,R\,i\,g\,h\,t\,\;A\,c\,t\,i\,v\,e\,\;V\,o\,x\,e\,l\,s\right)}$$

##### Lesion-adapted lateralization index (LALI)

In the LALI formula (see below) active voxels are also determined by counting those above the median *z* score within the ROI. However, in this method, the ROI is limited in each participant to only consider non-lesioned voxels. In other words, the size of the left sided ROI is decreased for each participant by the number of lesioned voxels that overlap with the ROI. In contrast, the right sided ROI is the same size as the ROI used for the SLI calculation. Activation in right and left ROIs is then expressed as a ratio of the number of active voxels compared to the total number of voxels in this limited, non-lesioned ROI. For example, if the ROI is 15274 voxels (i.e., anterior ROI used in this study), but for a given participant only 1000 voxels in the ROI are non-lesioned voxels, active voxels are quantified as the proportion of these 1000 voxels that were above the median *z*-score. This approach allows us to consider, in our population, a left-hemisphere ROI “highly active” if a large proportion of non-lesioned voxels are active, even if the participant has only a small amount of non-lesion tissue in the ROI.

$$\frac{((\frac{\#\,\;Left\,\;Active\,\;Voxels}{\#\,\;Left\,\;Nonlesioned\,\;Voxels})-(\frac{\#\,\;Right\,\;Active\,\;Voxels}{\#\,\;Right\,\;Nonlesioned\,\;Voxels}))}{((\frac{\#\,\;Left\,\;\,\;Voxels}{\#\,\;Left\,\;Nonlesioned\,\;Voxels})+(\frac{\#\,\;Right\,\;\,\;Voxels}{\#\,\;Right\,\;Nonlesioned\,\;Voxels}))}$$

#### Statistical Treatment

Spearman correlation coefficients and intraclass correlation coefficients (ICC) were calculated to determine the relationship of the LI values generated by the SLI and the LALI formulas.

Fisher’s exact tests were used to examine possible differences in language lateralization classification (left, right, bilateral) between SLI and LALI. A weighted Cohen’s Kappa provided a measure of agreement between SLI and LALI in the classification of language lateralization.

Lastly, we used Spearman correlation to examine the relationship between the difference in LI values produced by the LALI and SLI calculations (LALI - SLI) with (1) lesion size, (2) the percentage of lesion overlap in each ROI, (3) aphasia severity [i.e., Western Aphasia Battery-Revised Aphasia Quotient; WAB-R AQ (Kertesz, 2006)].

## Results

### LI Values Generated by the Two Methods

The LI values generated by the SLI and LALI formulas were highly correlated in the anterior (rho = 0.909; *p* < 0.001) and the posterior (rho = 0.895; *p* < 0.0001) ROIs. Further, the ICC suggests a high level of relatedness in the LI values generated by each method [anterior: 0.939; 95% CI (0.81 – 0.98); posterior: 0.943; 95% CI (0.83 – 0.98)].

### Language Lateralization Classification: Differences between SLI and LALI

Fisher’s exact test revealed a significant difference between the two calculation methods for language lateralization classification (i.e., left, right, bilateral), with two participants exhibiting a different classification in the anterior ROI (*p* = 0.004) and three in the posterior ROI (*p* = 0.028).

The weighted Kappa coefficient indicated only a moderate agreement of language lateralization classification for both the anterior ROI [*k* = 0.72; 95% CI (0.04 – 1.0)] and the posterior ROI [*k* = 0.60; 95% CI (0.24-0.96)].

### Individual Changes in Language Lateralization Classification

**FIGURE 4:**
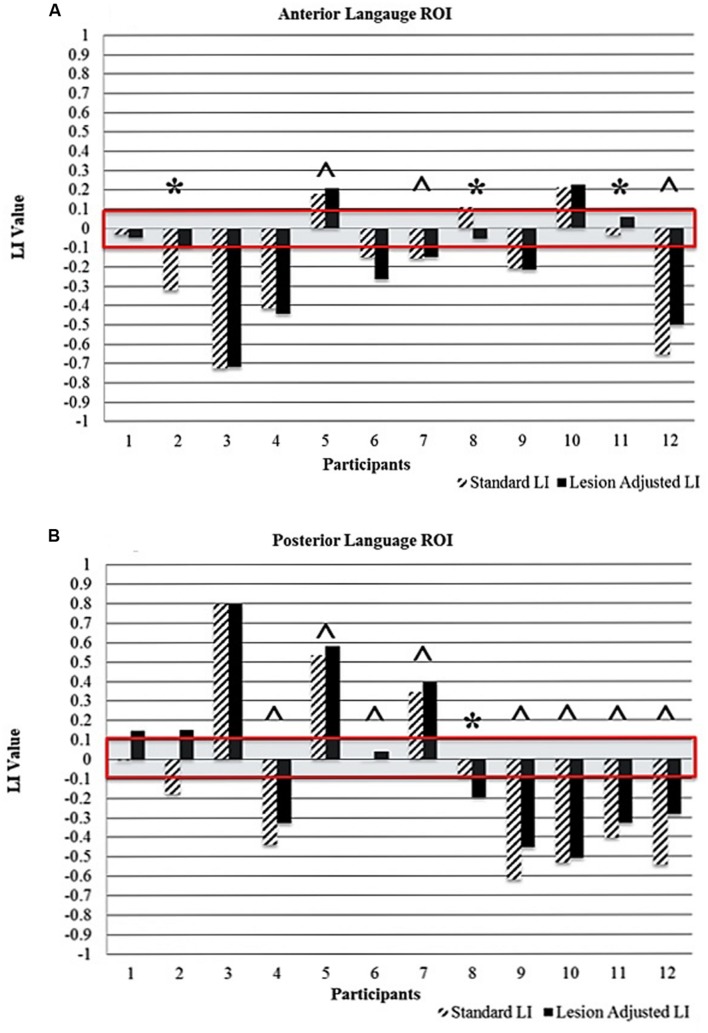
**Comparison of standard and lesion-adjusted language lateralization values (< -0.1 indicates right-lateralization and LI > 0.1 indicate left-lateralization) for the **(A)** anterior and **(B)** posterior language ROIs**. The red box highlights the bilateral language lateralization classification (-0.1 to +0.1). The ^∗^ indicates differences in either language lateralization classification and/or directionality of the LI value. The ˆ indicates a more leftward value with the LALI method, compared to the SLI method; however, language lateralization classification did not change.

**FIGURE 5:**
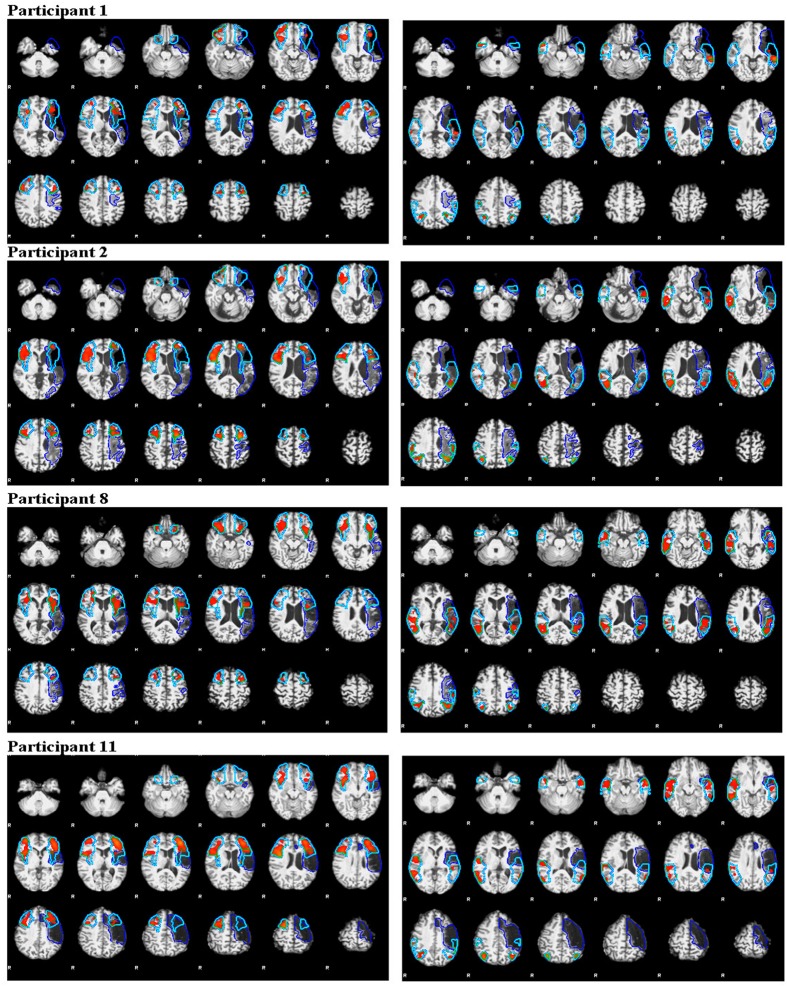
**Illustration of activation intensity (median *z* score) and lesion (dark blue outline) overlap with ROIs (turquoise outline) for participants who demonstrated different language lateralization classifications for the SLI and LALI calculation methods**. The anterior ROIs are depicted in the left panels and the posterior in the right panels.

**Figure 4A** (anterior ROI) and **Figure 4B** (posterior ROI) depicts each participant’s language lateralization values for both calculation methods. **Figure 5** illustrates the overlap of the lesion and language ROIs for the participants whose language classification (or LI directionality) differed with the application of the two LI calculation methods. The following sections describe the observed differences between the SLI and LALI calculation methods for each participant.

#### Anterior ROI

Using the SLI method, Participant 2 was classified as right lateralized (-0.3); whereas the LALI method classified him as bilateral (-0.09). For Participant 11, both methods yielded bilateral LI values; however, SLI method generated a more rightward (-0.03) value, and the LALI a more leftward (0.06) value. Although their language classification did not change, Participants 5, 7, and 12 demonstrated more leftward LI values using the lesion-adjusted formula, as compared to the standard calculation method. Participant 8 was classified as bilateral by both methods; however, the SLI yielded a more leftward value (0.1) and the LALI method, a more rightward LI value (-0.05). Likewise, a (slightly) more rightward value resulted from LALI method for Participants 4, and 6; however, they did not change classification (see **Figure 4A**).

#### Posterior ROI

For Participant 1, the SLI method produced a bilateral classification (-0.01), while the LALI returned values indicating left lateralization (0.13). Using the SLI method, Participant 2 was classified as right lateralized (-0.2); whereas the LALI method classified him as bilateral (-0.1). Further, compared to the SLI formula, the LALI method generated more leftward LI values for Participants 4, 5, 6, 7, 9, 10, 11, and 12; however, language classification did not change. For participant 8, the SLI was bilateral (-0.07) and the LALI was right lateralized (-0.2) (see **Figure 4B**).

### Relationship of the Difference between SLI and LALI Values with Lesion Size and Lesion/ROI Overlap

**FIGURE 6:**
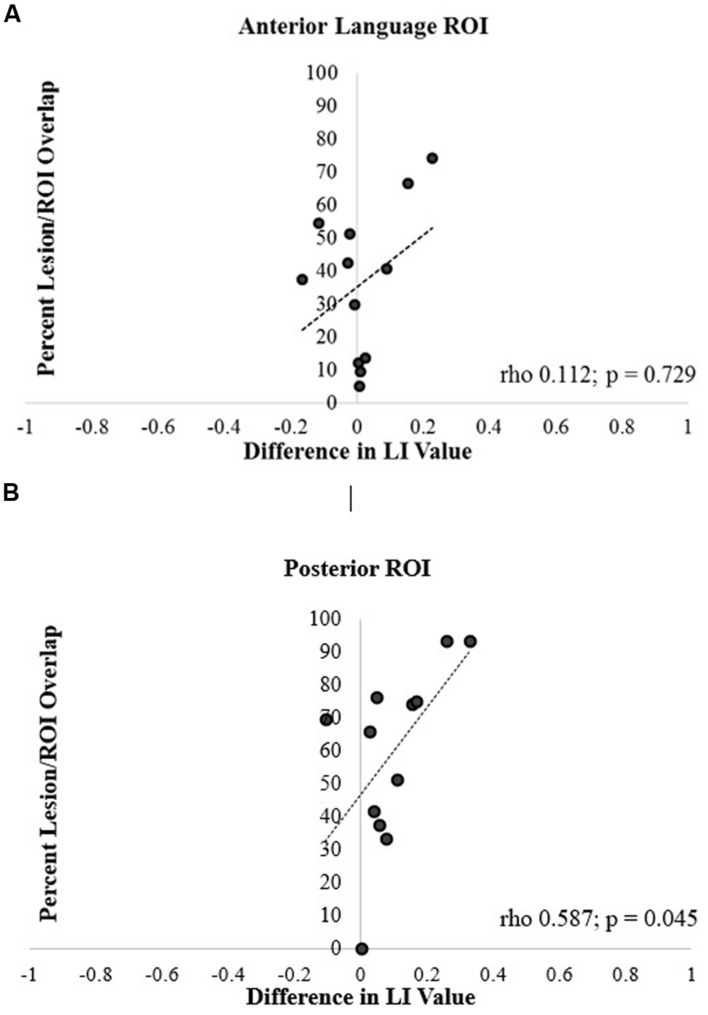
**Correlation of percentage of lesion and ROI overlap with difference in language lateralization index (LI) values for the (A) anterior and (B) posterior language ROIs**.

For the anterior ROI, there was no significant relationship between lesion/ROI overlap and the difference between LALI and SLI values (rho = 0.112, *p* = 0.729; **Figure 6A**). However, for the posterior ROI, for participants that had a greater overlap between the ROI and the lesion, there was a greater difference between the results of two formulas (posterior ROI: rho = 0.587, *p* = 0.045; **Figure 6B**); with the SLI method biasing values rightward.

**FIGURE 7:**
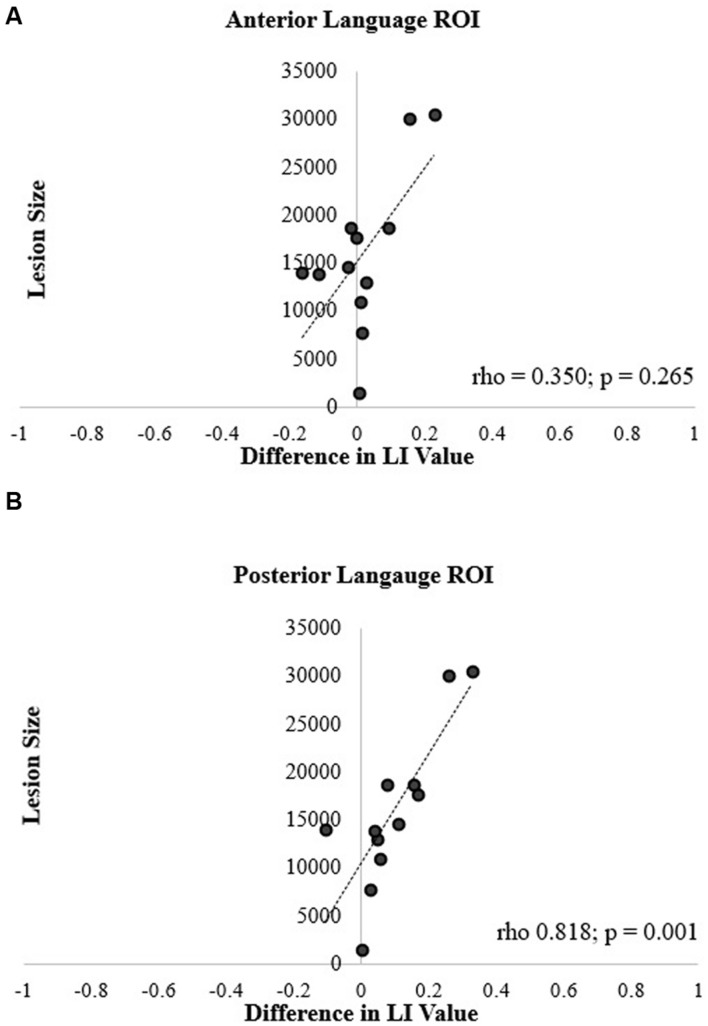
**Correlation of lesion size and difference in language LI values for the (A) anterior and (B) posterior language ROIs**.

Spearman correlation coefficients revealed no significant correlation between lesion size and the difference in LI values between the two methods for the anterior ROI (rho = 0.350, *p* = 0.265; **Figure 7A**). In contrast, the correlation was significant in the posterior ROI (rho = 0.818, *p* = 0.001; **Figure 7B**). In essence, in participants with larger lesions, there was a greater difference between the LI values generated by two calculation methods in the posterior ROI.

### Correlation of Aphasia Severity with SLI and LALI

For both ROIs, Spearman correlation revealed no significant relationship between aphasia severity (i.e., WAB-R AQ) and (1) LI calculation method, (2) lesion size, or (3) lesion/ROI overlap.

## Discussion

We examined whether application of a SLI and an LALI calculation method, in two language ROIs during a verb generation task, generated differences in language lateralization classification and LI values. Further, we examined the relationship between LALI and SLI values with left-hemisphere lesion size and percent overlap between ROI and lesion. Lastly, we asked if aphasia severity influenced the LI values for either calculation method. To our knowledge, this is the first study to document the differences in LI values based on standard and lesion-adjusted methods for calculating language lateralization indices in people with post-stroke aphasia. Although LI values generated by the two approaches were highly correlated, the results confirmed our hypothesis that the two methods produced different lateralization classifications. Further, the lesion size (both ROIs) and degree of lesion overlap with the ROI positively correlated with the difference in LI values, at least for the posterior ROI. However, in this sample, there was no relationship observed between aphasia severity and LI values for either the SLI or the LALI. These findings highlight several points of consideration when calculating and interpreting language lateralization indices in people with post-stroke aphasia.

### Factors that Influence LI Calculation Outcomes in People with Aphasia

#### Lesion Masking in Stroke Research

**FIGURE 8:**
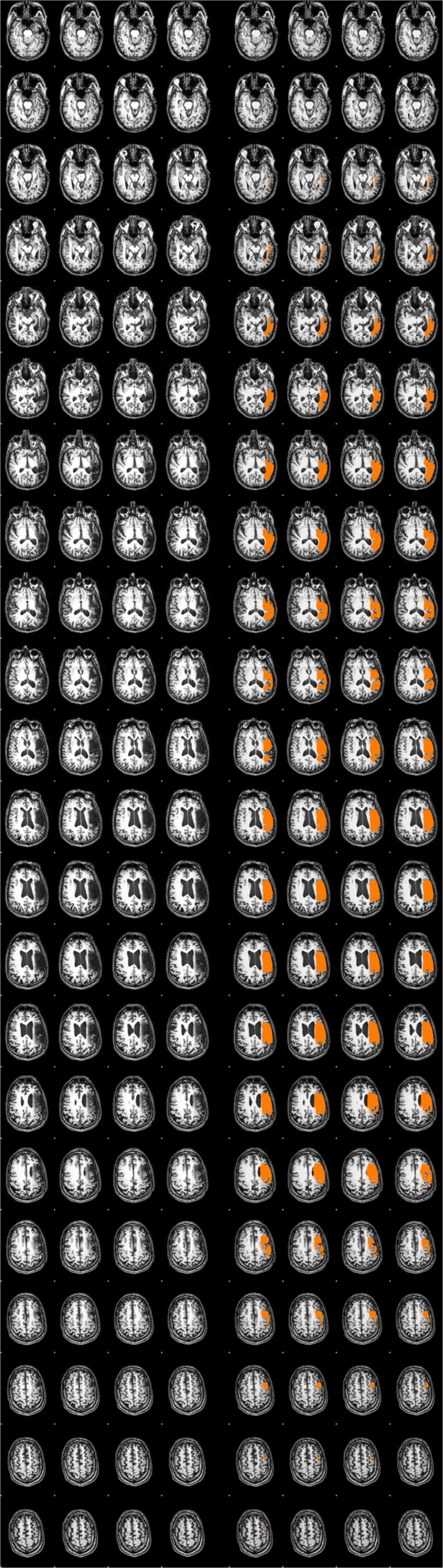
**Side-by-side comparison of Participant 8’s T1-weighted MRI scan, with and without the lesion mask, revealing viable tissue in the both ROIs**.

Creation of lesion masks is a critical step in the post-processing of fMRI data in people with post-stroke aphasia (Crinion et al., 2013). The literature reveals common procedures such as neuroanatomists hand-tracing lesion-masking using the T1-weighted MRI scan (Allendorfer et al., 2012a; Fridriksson et al., 2012); with a more recent movement toward automated lesion masking processes (Ripollés et al., 2012), and Gaussian naïve Bayes classification of lesions (Griffis et al., 2016). However, it is likely that the extent of the lesion cannot be fully discerned using the T1-weighted MRI scan, which may influence accurate calculation of LI and subsequent language lateralization classification. For example, in the current study, Participant 8 appears to have healthy tissue included the lesioned mask that overlaps the left anterior and posterior ROIs (see **Figure 8**). His LI values revealed a pattern opposite to our hypothesized direction (i.e., the LALI, not the SLI, biased the LI values rightward). At least part of the anterior ROI encompasses what appears to be residual tissue that has been assigned to the lesion. Similarly, in the posterior language area, there are some small islands of what appear to be relatively normal looking brain tissue that is included in the lesion mask. Inclusion of viable tissue may have occurred due to the difficulty in registering the depth of the lesion using a T1-weighted scan; in other words, the tissue is likely lesioned, but not as deeply as it was more anteriorly. This speaks supports the notion additional measures may be necessary to fully identify the extent of the lesion versus viable tissue (i.e., via diffusion-weighted imaging, perfusion-weighted image, etc.) (Fridriksson et al., 2006).

#### Bilateral Language Classification

For the current study, we defined language as bilateral for LI values between -0.1 and 0.1; while other researchers (Binder et al., 1996; Springer et al., 1999) have categorized language as symmetrical for values between -0.2 and 0.2. On the surface, it seems the definition of bilateral, or symmetrical, language could influence the differences observed between the SLI and LALI observed in this project. For example, for the posterior ROI, Participants 2 and 8 would not have been classified differently using the SLI and LALI calculation methods if, we had defined bilateral representation as -0.2–2.0. The differences for these seemingly conflicting language lateralization classification schemas can be attributed to the different fMRI tasks employed. Binder et al. (1996) and Springer et al. (1999) report fMRI results based on a semantic decision vs. a tone decision task, which generates a strong leftward lateralization response. The verb generation task, as used in the current study, typically generates a less strongly lateralized pattern of activation; thereby necessitating a smaller interval to define bilateral (Szaflarski et al., 2008). The difference between these two tasks regarding the degree of evoked language lateralization is likely due to the two things (Szaflarski et al., 2008). First, the semantic decision versus tone decision allows researchers to control for motor responses, auditory processing, attention, and working memory. By comparison, the verb generation task is not designed to control for attention and working memory. Instead, this task is able to tease-apart articulatory/motor aspects, noun-verb semantic association, and auditory noun comprehension [as described in section “Event-Related Verb Generation Task (ER-VGT)” of the Method] (Szaflarski et al., 2006, 2008, 2014; Allendorfer et al., 2012a,b). Going forward, it will be important to understand how different tasks, and subsequent varying definitions of bilateral, may relate to the use of a lesion-adjusted formula to determine language lateralization.

#### The Role of Lesion Size and Lesion/ROI Overlap

The inconsistent differences between the LI values resulting from the two methods seems to be at least partially explained by relationship of lesion size and lesion/ROI overlap. Moderate to strong correlations were observed for lesion size and lesion/ROI overlap with the difference between the two calculation methods. However, this outcome was only observed in the posterior ROI. Since the verb generation task engages both receptive (comprehending the stimulus noun) and expressive language components, we would expect that a lesion in either ROI would have an impact on the calculation of LI using the lesion-adjusted approach. As such, the observed significant correlations in the posterior ROI are not surprising. The lesion/ROI overlap (or lack thereof) may explain the non-significant correlations observed for the anterior ROI. A review of the data (**Figure 3**) revealed that four participants had lesions that overlapped with only 15% of the anterior ROI. In contrast, the lesion overlapped with the posterior ROI by at least 38% for all participants. At the group level, the mean difference of the ROI/lesion overlap is significantly lower (*p* = 0.009) in the anterior ROI (*M* = 36.39, *SD* = 22.98), as compared to the posterior ROI (*M* = 59.44, *SD* = 27.41). These findings suggest that a minimum level of overlap is required to affect results when using a SLI versus an LALI method. It is likely the case that lesion/ROI overlap only matters when key lesioned voxels are included in the overlapping region. Accordingly, the larger the degree of overlap, the more likely key lesioned voxels are included in the overlap. A recent proliferation of voxel-based lesion symptom mapping (VLSM) offers support for this theory. A VLSM analysis provides detailed information regarding the relation between behavioral performance and the lesion location by statistically testing whether people with specific lesion locations are more likely to have impairments on a given task (Tyler et al., 2005). Researchers have used VLSM analyses to predict how well lesion location predicts a multitude of behavioral measures, including performance on speech fluency (Fridriksson et al., 2013), semantic naming errors (Walker et al., 2011), and sentence production (Faroqi-Shah et al., 2014), just to name a few. This work has also been applied to post-stroke language recovery research to determine how treatment “responders” differ from “non-responders.” For example, people with aphasia tended to respond more favorably to an anomia treatment when areas typically associated with lexical retrieval and phonological process (i.e., Brodmann’s areas 37 and 39) were intact (Fridriksson, 2010).

## Conclusion: Importance of Quantifying Hemispheric Contribution to the LI

The two LI methods generated significant differences in either language lateralization classification and/or directionality of the LI value in at least one ROI, for four participants (30% of the sample). Upon closer examination, three of the four participants’ LI values were biased rightward with the SLI method. The fourth participant’s unexpected rightward bias using the LALI (Participant 8) may be explained by the aforementioned lesion-masking challenges. Further, 10 participants demonstrated a more leftward LI with application of the LALI formula in either the posterior ROI (*n* = 7) or the anterior ROI (*n* = 3). Although not definitive, these results suggest that the SLI calculation method may bias the language lateralization classification, and thus skew interpretation of data if the lesion is not accounted for in the LI formula (especially in smaller samples). This issue becomes especially critical as, we seek to refine our understanding of the important, yet distinct contributions of the left and right hemisphere in post-stroke language recovery.

This study represents and initial effort to determine whether a lesion-adjusted and a SLI calculation method produce different language lateralization indices, and determine underlying relationships between lesion size and lesion/ROI overlap. It is premature to suggest that the LALI method has utility in predicting behavioral performance (and for which language tasks it might be most useful). This point is underscored by the non-significant correlation between aphasia severity and language lateralization indices (for both calculation methods) observed in the current study. It is logical to assume that aphasia severity could impact the outcomes of a LI calculation, irrespective of lesion overlap or lesion size; however, this did not bear out in the exploratory analyses (likely due to the small sample with a restricted range of severity levels). Nonetheless, the data suggest that continued examination of the factors described in this paper is warranted. Going forward, it will be important to examine the relationship of lateralization indices generated by SLI and LALI methods with in-scanner behavioral performance and comparable behavioral tasks outside of the scanner. Accordingly, it is also essential to examine the differences, if any, across various fMRI language paradigms. Another factor that must be considered is whether differences exists between SLI and LALI calculation methods in terms of assessing treatment-induced changes in language reorganization. A final issue to consider is the various ways LALIs might be derived (Bonakdarpour et al., 2007; Sebastian and Kiran, 2011); the current paper only examined one method. Valid and reliable examination of these variables requires larger sample sizes, which may be facilitated by collaborative efforts.

A better understanding of how, and perhaps more importantly, when (perhaps with a certain threshold of lesion/ROI overlap), to apply a SLI or an LALI calculation method in the study of aphasia would increase reproducibility of results, and perhaps, help to resolve some of the lingering debate regarding the respective role of each hemisphere in language processing and post-stroke recovery. Deeper examination of the issues raised in the current study, together with VLSM can also aid in the development of more efficacious application of multimodal interventions. More frequently, researchers are combining biological treatments such as transcranial magnetic stimulation (TMS) or transcranial direct current stimulation (tDCS) with traditional behavioral interventions (Berthier et al., 2011; Allendorfer et al., 2012a; Holland and Crinion, 2012; Faseyitan et al., 2013; Shah et al., 2013). In order to positively influence clinical practice and language recovery in post-stroke aphasia, clarity is needed regarding the underlying mechanism, we aim to stimulate (or inhibit) with these biological treatments, and thereby boost outcomes of behavioral interventions. For these reasons, it is important for researchers to consider the implications of different LI calculation methods for people with stroke-induced aphasia.

## Author Contributions

AD made substantial contributions to conception and the design of the work; she was the primary investigator on the grants. She also collected and interpreted the data for the paper and drafted the paper for critical review by the authors listed below. She agrees to be accountable for all aspects of the work. JV made substantial contributions to the conception and design of the work, contributed to the interpretation of the data, critically revised the manuscript, approved the final version of the paper. She agrees to be accountable for all aspects of the work. She served as AD’s neuroimaging mentor on the grant. TM made substantial contributions to the conception and design of the work, contributed to the interpretation of the data, critically revised the manuscript, approved the final version of the paper. He agrees to be accountable for all aspects of the work. MA made substantial contributions to the conception and design of the work, contributed to the interpretation of the data, critically revised the manuscript, approved the final version of the paper. He agrees to be accountable for all aspects of the work. JS made substantial contributions to the conception and design of the work, contributed to the interpretation of the data, critically revised the manuscript, approved the final version of the paper. He agrees to be accountable for all aspects of the work. He served as AD’s neuroimaging mentor on the grant. SH made substantial contributions to the conception and design of the work, contributed to the interpretation of the data, critically revised the manuscript, and approved the final version of the paper. He agrees to be accountable for all aspects of the work. He served as AD’s neuroimaging mentor on the grant.

## Conflict of Interest Statement

The authors declare that the research was conducted in the absence of any commercial or financial relationships that could be construed as a potential conflict of interest.

## Footnotes

1 http://www.empirisoft.com/
